# Organic matter decay and bacterial community succession in mangroves under simulated climate change scenarios

**DOI:** 10.1007/s42770-024-01455-2

**Published:** 2024-07-19

**Authors:** Juanita H. Solano, Marta A. Moitinho, Josiane B. Chiaramonte, Laura Bononi, Ana Paula Packer, Itamar S. Melo, Francisco Dini-Andreote, Siu Mui Tsai, Rodrigo G. Taketani

**Affiliations:** 1 Brazilian Agricultural. Research Corporation, Embrapa Environment, SP 340. Highway—Km 127.5, Jaguariúna, SP 13820-000 Brazil; 2 College of Agriculture Luiz de Queiroz, University of São Paulo, Pádua Dias Avenue, 11, Piracicaba, SP 13418-900 Brazil; 3 Department of Plant Science and Huck Institutes of the Life Sciences, The Pennsylvania State University, University Park, PA USA; 4 Center for Nuclear Energy in Agriculture, University of São Paulo, Piracicaba, SP Brazil; 5 Centre for Mineral Technology, CETEM, MCTIC Ministry of Science, Technology, Innovation and Communication, Av. Pedro Calmon, 900, Cidade Universitária, Ilha do Fundão, Rio de Janeiro, 21941-908 Brazil; 6 Sustainable Agriculture Sciences, Rothamsted Research, West Common, Harpenden, AL5 2JQ UK

**Keywords:** Bacterial community, Mangrove plant degradation, Succession, Microcosms, Climate change

## Abstract

Mangroves are coastal environments that provide resources for adjacent ecosystems due to their high productivity, organic matter decomposition, and carbon cycling by microbial communities in sediments. Since the industrial revolution, the increase of Greenhouse Gases (GHG) released due to fossil fuel burning led to many environmental abnormalities such as an increase in average temperature and ocean acidification. Based on the hypothesis that climate change modifies the microbial diversity associated with decaying organic matter in mangrove sediments, this study aimed to evaluate the microbial diversity under simulated climate change conditions during the litter decomposition process and the emission of GHG. Thus, microcosms containing organic matter from the three main plant species found in mangroves throughout the State of São Paulo, Brazil (*Rhizophora mangle*, *Laguncularia racemosa*, and *Avicennia schaueriana*) were incubated simulating climate changes (increase in temperature and pH). The decay rate was higher in the first seven days of incubation, but the differences between the simulated treatments were minor. GHG fluxes were higher in the first ten days and higher in samples under increased temperature. The variation in time resulted in substantial impacts on α-diversity and community composition, initially with a greater abundance of Gammaproteobacteria for all plant species despite the climate conditions variations. The PCoA analysis reveals the chronological sequence in β-diversity, indicating the increase of Deltaproteobacteria at the end of the process. The GHG emission varied in function of the organic matter source with an increase due to the elevated temperature, concurrent with the rise in the Deltaproteobacteria population. Thus, these results indicate that under the expected climate change scenario for the end of the century, the decomposition rate and GHG emissions will be potentially higher, leading to a harmful feedback loop of GHG production. This process can happen independently of an impact on the bacterial community structure due to these changes.

**Supplementary Information:**

The online version contains supplementary material available at 10.1007/s42770-024-01455-2.

## Introduction

Mangrove forests are coastal tropical ecosystems located in the transition between sea and continent. They are highly productive serving as both sources and sinks for substantial quantities of organic matter [1, 2]. Notably, a significant proportion of the carbon generated by the vegetation becomes sequestered in the sedimentary strata, where the prevailing anaerobic conditions facilitate its accumulation [3]. The balance between production and decomposition is vital for maintaining the carbon cycle in this ecosystem. However, this environment is currently threatened by human activities (e.g. urban development, aquaculture, and industrial waste) and climate changes which could perturb the delicate equilibrium of the decomposition process and, consequently, imperil the integrity of these vital coastal ecosystems [1, 4].

Mangroves are considered the leading producers of greenhouse gases (GHG) in coastal areas. Microorganisms play a central role in the emissions from these sediments, as they are responsible for crucial biogeochemical processes such as methanogenesis (CH_4_) and denitrification (N_2_O) [5, 6]. However, there is insufficient data about the production of N_2_O and CH_4_ there. The anaerobic nature of mangrove sediments, the high productivity, and intense microbial activity contribute to these emissions [5, 7]. Furthermore, several investigations have unveiled that GHG are intrinsically linked to ambient environmental conditions, underlining the need for a more comprehensive understanding of the repercussions of climate change upon this process [5, 8, 9]. Recently, it was shown that the increase in sediment temperature increases methane emissions in mangrove sediments which would lead to an overall yearly increase in GHG emissions in mangroves [10].

According to several studies, global climate change will increase marine water temperature, and the increase in atmospheric CO_2_ will lead to the acidification of marine water [11]. However, the effects of these processes are hard to predict as they can act as positive or negative feedbacks [2, 12]. This complexity is caused by the different effects of temperature and pH on each microbial population that inhabits any ecosystem. This intricate web of interactions is further compounded by the multifaceted responses of individual microbial populations residing within mangrove ecosystems to the varying dimensions of temperature, pH, and salinity, all of which fluctuate significantly due to the influence of rain-induced freshwater inflow [13]. Thus, the adaptability of these microbial communities to changes in the pivotal environmental variables, including temperature and pH, could significantly shape the patterns of GHG production.

The decomposition of organic matter produced in mangroves is carried out by a complex community of microorganisms involving fungal and bacterial populations [14]. In addition, the process of degradation and succession is influenced by local environmental characteristics and plant species [3, 15, 16]. Thus, the response of these communities to changes in critical environmental factors such as temperature and pH might affect the production of GHG.

Therefore, this study was designed to evaluate the hypothesis that (1) the increase in temperature and pH predicted for the end of the century will increase the rate of decomposition of different plant species litter, and (2) the environmental changes would affect the succession process of the bacterial community. Thus, we applied GHG emissions quantification and metabarcoding to study the community composition and activity in mangrove sediment microcosms.

## Materials and methods

### Sample aquisition

To understand the effects of the increase in temperature and ocean acidification in the decomposition of plant litter in mangrove samples, we collected sediments, leaves and local estuarine water to construct microcosm experiments. The choice to use microcosms to simulate changes in temperature and pH was done to reduce the variation of other environmental parameters, such as seasonal climate variation and tidal action. Microcosms also allow easier tests of hypothesis which could be complex to test in the natural environment, however, because microcosms are a simplification of what is found in situ the true response may differ from what is seen in natural environments. Nevertheless, they are valuable tools for understanding factors separately and narrowing their influences. Thus, microcosms are important tools for studying complex ecological questions including climate change [17] The collection of plant, sediment and water was approved by the Biodiversity Authorization and Information System (#65580) and the study complies with local and national guidelines. Samples were collected in a mangrove located in the city of Cananéia in São Paulo state (Brazil) (25°05’01.8” S– 47°57’45.7” W). Fresh and healthy leaves from the three tree species found in this mangrove (*Rhizophora mangle*, *Laguncularia racemosa*, and *Avicennia schaueriana*) were collected and placed in sterile bags (more details on sampling are described in reference 15). These plants are the only tree species present in this forest, have contrasting leaf chemistry, and are unevenly spread in the mangrove [16, 18]. Plant species identification was made by Juanita H. Solano and Rodrigo G. Taketani based on marked morphological differences between the species. Sediments from the top 0–10 cm layer were sampled with a sterile spade and placed in a sterile bag. Brackish water was sampled in 1 L sterile glass bottles. Samples were collected from four locations on the sampled mangrove. Samples were kept in ice until arrival in the laboratory within 24 h, where they were immediately processed.

### Microcosm experiment

Microcosms were constructed in sealed 500 mL polypropylene bottles with a polycarbonate three-way stopcock valve for the GHG quantification. Each microcosm consisted of 100 g of sediment topped with 16 discs (1.7 cm diameter) of leaves from a single mangrove species (*Rhizophora mangle*, *Laguncularia racemosa*, or *Avicennia schaueriana*) and 10 ml of sampled water (figure S1).

According to the prediction of IPCC2014 [19] for the region, the average temperature will rise by 2 °C (from 27.5 °C to 29.5 °C), and pH will go from 7.05 to 6.74. Thus, for each plant species, four treatments were set to contrast the current temperature (T1) and pH (pH1) with the predicted increased temperature (T2) and lower (acidified) pH (pH2). Thus, the treatments were named T1pH1, T1pH2, T2pH1, and T2pH2, where T1pH1 had the current conditions and can be considered the control and T2pH2 had the predicted values for 2100 IPCC2014 [19]. The temperature was maintained constant throughout the experiment in incubators, and the pH was adjusted with HCl 1M [20, 21]. Microcosms were destructive, set-in quadruplicates, and samples were taken at 3, 7, 15, 30, and 45 days.

### Determination of organic matter decomposition

The decomposition rate of plant organic matter was determined by comparing the dry leaf disk weight before the experiment and the dry weight in each decomposition stage. To determine the dry weight, leaves were rinsed with sterile distilled water to remove sediment and set to dry at 45 °C for 72 h. The rate of decomposition (*k* constant) was determined at each sampling date, according to Olson [22]. The initial weight of each disk was considered as the average weight of ten leaf disks. Using the *k* constant, we were able to calculate the time to decompose 50% of the plant material (t_50_) according to Olson (1963).

### Greenhouse gas quantification

Measurements of GHG were made every day for the first eight days and every other day after that. Samples were taken from the three-way valve with a 20 mL sterile syringe. Samples were taken at 0, 5, and 10 min to evaluate the gas flux. GHG were quantified in a TRACE 1310 (Thermo Scientific) chromatograph with a (TriPlus RSH) automatic injector. An electron capture detector was used for N_2_O quantification and a flame ionization detector for CO_2_ and CH_4_. A Hayesep Q^®^ separation column was used at 100 °C and helium was used as the carrier gas. Gas flow was calculated as described previously [23]. N_2_O and CH_4_ were converted to CO_2_ equivalents as described previously [19].

### Sediment DNA extraction and sequencing

DNA present in 0.25 g of decomposing leaf disks was extracted using the DNA Extraction Kit - DNA Power Soil™ (MoBio Laboratories, Carlsbad, CA, USA) following the manufacturer’s protocol. The amplification of the V6 region of the 16 S rRNA gene was performed using primers 967 F [24] and 1195R [25]. PCR [24] and sequencing [15, 18] conditions were described previously. DNA sequences were analyzed using Qiime [26] as described in the Brazilian Microbiome Project guidelines (https://www.brmicrobiome.org/clusteringmeth). Samples were rarefied to the lowest sequence count (14,000) and further analyzed in Qiime and phyloseq [27]. Sequences are available on https://www.mg-rast.org/linkin.cgi?project=mgp97665.

### Data analysis

All statistical analysis was performed in the R environment using RStudio. Analysis of variance (ANOVA) followed by Tukey HSD was performed with the package *agricolae*. All community diversity analysis was performed with the *phyloseq* and *vegan* packages [27, 28].

## Results

### Degradation of plant material

**Fig. 1:**
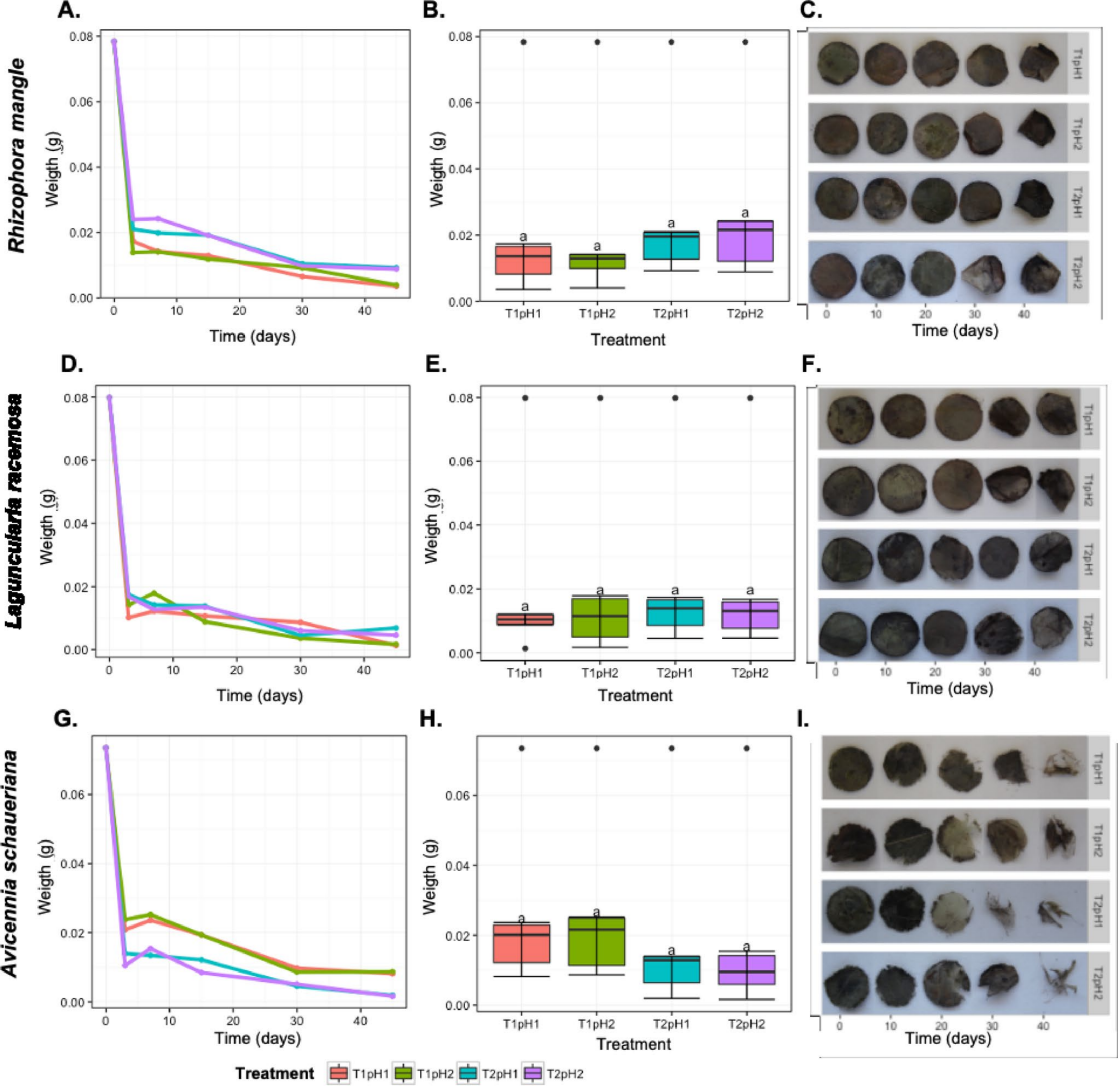
Dry weight decay of the mangrove plant material. **A**,**D**, **G** - Average dry weight decay over time **B**, **E**, **H**– Average decay along each treatment. **C**, **F**, **I**– Photographic example of a representative decomposed leaf. Letters over each treatment represent significant differences between treatments according to the Tukey test and an α of 0.05

The analysis of the remaining weight of the plant material showed that the rate of decay of each plant material was different (Fig. 1). *Laguncularia racemosa* had the lowest decomposition rate (65.9%), followed by *Rhizophora mangle* (71.8%) and *Avicennia schaueriana* (74.1%). However, the rate of degradation and half-life followed the same pattern (Table 1), indicating that *L. racemosa* was more resistant to the degradation. The weight loss pattern was similar for all the plant species, i.e., most of the weight loss was observed in the first seven days (Fig. 1A, D, G). This observation is caused by the loss of soluble substances (e.g., carbohydrates and proteins) and the degradation of these molecules.

**Table 1:** Comparison of the constant of decomposition (*k*) and the half-life (t_50%_) of the leaf litter from *Avicennia Schaueriana*,*Rhizophora mangle* and *Laguncularia racemosa* in microcosms of mangrove sediments during leaf litter during 45 days of degradation. Letters over each treatment represent significant differences between treatments according to the Tukey test and an α of 0.05

Species	k (g.g^− 1^.day^− 1^)	t_50%(days)_
*A. schaueriana*	0.22^a^	4.4^B^
*R. mangle*	0.20^a^	5.7^B^
*L. racemosa*	0.16^b^	7.4 ^A^

The decomposition of *R. mangle* and *A. schaueriana* showed a minor effect of the increased temperature (Fig. 1A, B, G, H). In addition, the degradation of *Rhizophora* slower in the samples with increased temperature (T2) while *Avicennia* was faster. This pattern, however, was not confirmed by ANOVA.

### Greenhouse gases emissions

**Fig. 2:**
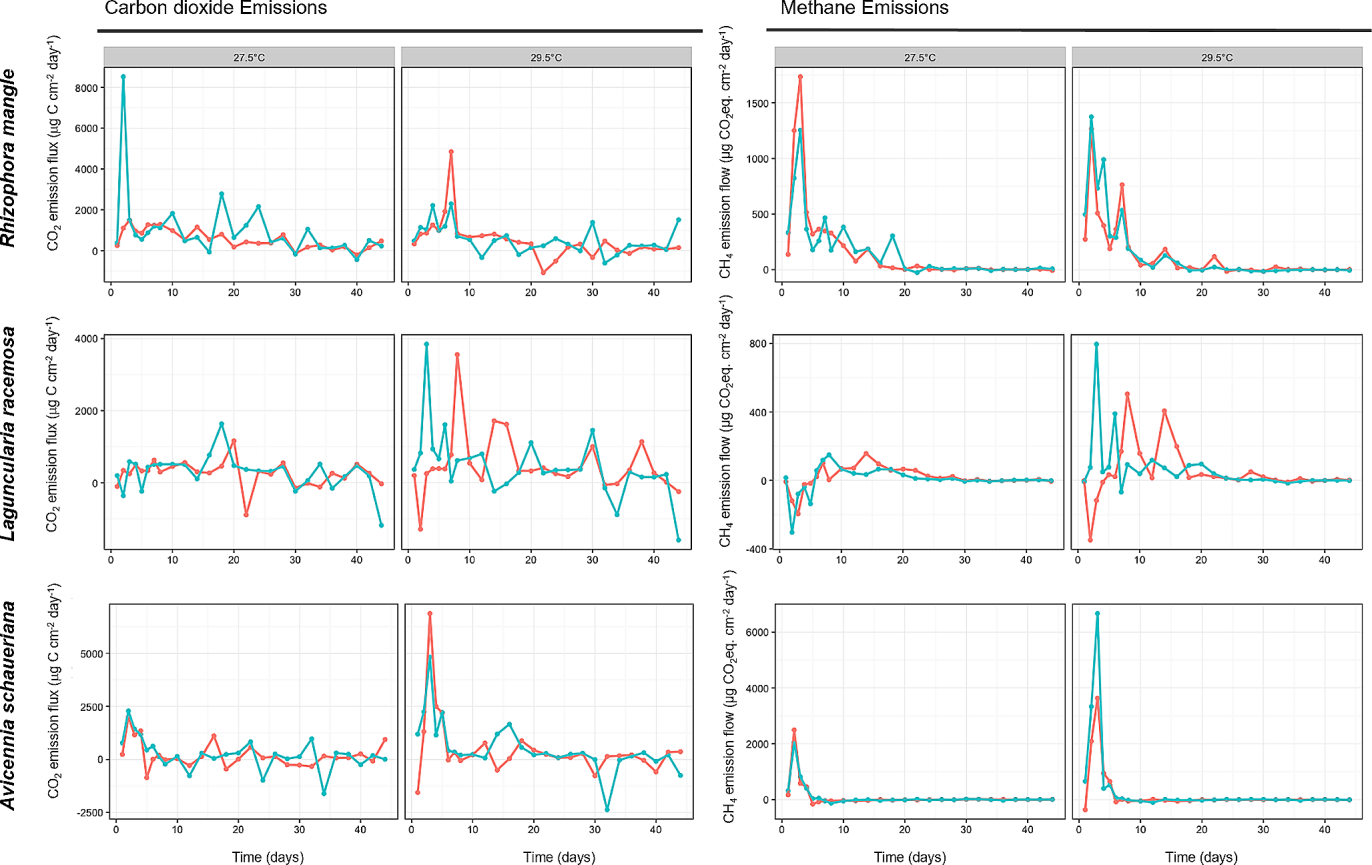
Flux of carbon dioxide and methane emissions in microcosms of mangrove sediments during leaf litter decomposition. Blue lines indicate the average emission in acidified pH (6.74), and Red lines in current pH (7.05)

The CO_2_ flux of *R. mangle* was the highest between the evaluated microcosms (701.82 µg C cm^− 2^ day^− 1^), followed by *A. schaueriana* (399.73 µg C cm^− 2^ day^− 1^) and *L. racemosa* (382.64 µg C cm^− 2^ day^− 1^) (Fig. 2). Only the microcosms of *Rhizophora* presented a significantly different emission of CO_2_ according to Tukey’s HSD.

The flux of CO_2_ was higher in samples under increased temperature (29.5 °C) during the first ten days of incubation (Fig. 2). This peak in the emissions translated into a higher average in these microcosms (table S1), except for the *R. mangle* microcosms. The acidification produced a similar effect, leading to the increase in the average flow of CO_2_. After the first ten days, the fluxes were constant, and the variation among samples made it hard to distinguish between treatments.

The flux of CH_4_ also varied significantly between leaf sources. The microcosms containing leaves of *A. shaueriana* and *R. mangle* produced the highest emission fluxes. (235.75 and 193.87 µg CO_2_eq cm^− 2^ day^− 1^, respectively), in contrast to the *L. racemosa* average (34.54 µg CO_2_eq cm^− 2^ day^− 1^) and that of the control (Fig. 2 and table S1). Parallel to the carbon dioxide fluxes, the methane fluxes were higher in the first ten days in samples under increased temperature (Fig. 2). The effect of the increased temperature was a significant rise in the average flux of emission in *L. racemosa* and *A. schaueriana* microcosms (table S1).

The emissions of N_2_O were low, and the variation within samples higher than between samples which hindered comparisons between treatments.

### Bacterial community associated with decomposing leaves

The bacterial community associated with the decomposing material was evaluated using 16 S rRNA sequencing. This showed no significant differences in the α-diversity between the different plant materials (figure S2). However, when each plant was analysed separately, the effect of time was significant in the Observed OTUs (S_obs_) (but not for other metrics) according to ANOVA in *R. mangle* and *A. shaueriana*. However, for *L. racemosa*, time and temperature were significant for Shannon’s index, and the interaction between time and temperature was also significant for S_obs_, CHAO1 and Faith’s PD.

These communities were mainly composed of Proteobacteria (Gammaproteobacteria, Deltaproteobacteria, Alphaproteobacteria, respectively), Firmicutes, Actinobacteria, Chloroflexi and Bacteroidetes (fig. S3). However, along with the experiment, several changes were observed in these communities. In *R. mangle*, there is a decrease in Gammaproteobacteria (66.86–38.95%) and an increase in Deltaproteobacteria (5.71–24.89%) and Anaerolineae (0.2–3.60%) (fig. S3). In *L. racemosa*, there was not only a decrease in Gammaproteobacteria (50.64–25.49%) but in Epsilonproteobacteria (5.86–1.28%) accompanied by an increase in Deltaproteobacteria (14.81–33.96%) and Acidimicrobiia (0.58–1.50%) (fig. S3). While in *A. schaueriana*, the decrease in Gammaproteobacteria (51.63–29.46%) and Clostridia (21.64–2.42%) occurred with an increase in Deltaproteobacteria (4.89–25.91%), Alphaproteobacteria (2.47–8.84%) and Anaerolineae (0.20–6.74%) (fig. S3).

**Fig. 3:**
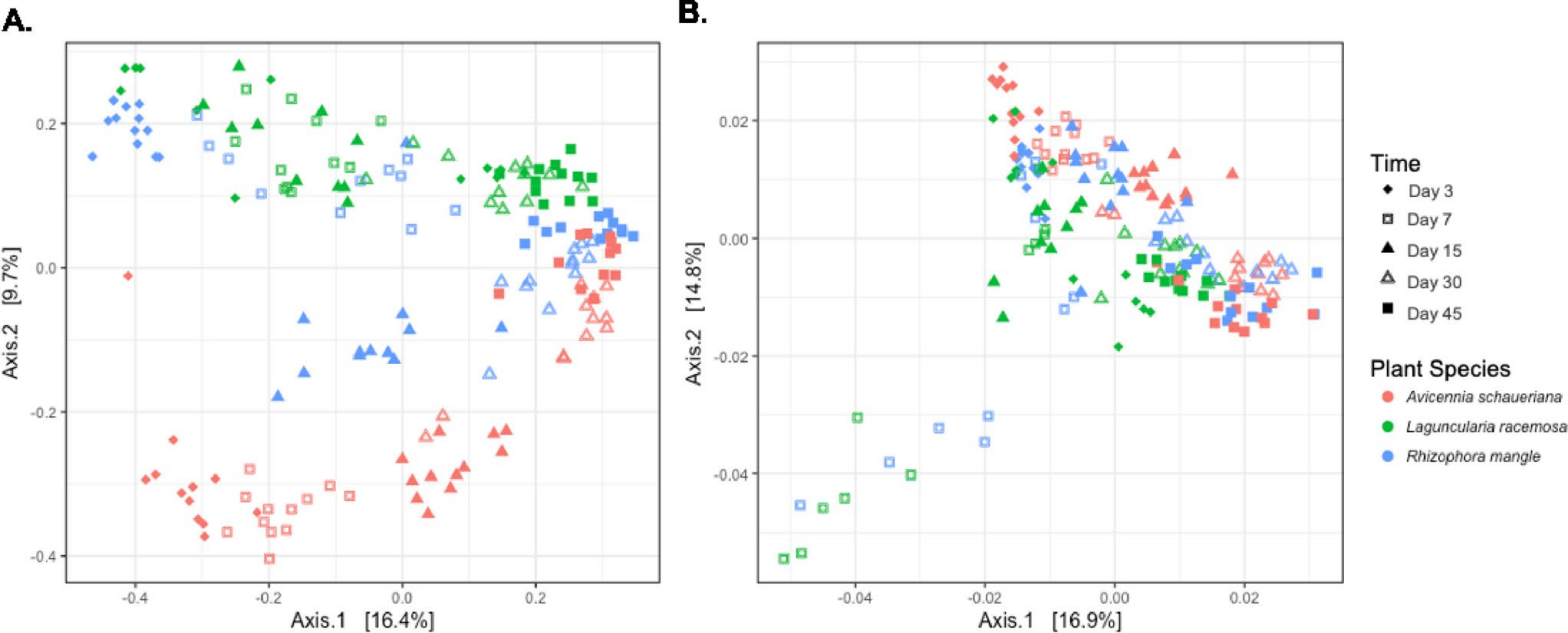
Principal coordinate analysis (PCoA) in microcosms of mangrove sediments during leaf litter decomposition. (**a.**) PCoA based on Bray-Curtis distance matrix; (**b.**) PCoA based on *weighted*-UniFrac distance matrix

The principal coordinate analysis (PCoA) based on Bray-Curtis of the bacterial communities found in the decaying leaves indicated that these samples segregate based on plant species and time (Fig. 3). The PCoA based on the Bray-Curtis distance matrix showed that samples from the beginning of the experiment were more different from each other than those samples from the end (45 days). This effect was not observed in the PCoA from weighted UniFrac. This PCoA showed that the samples from day 3 were similar. On day 7, samples from *Laguncularia* and *Rhizophora* separated from the *Avicennia*. In all latter samples, the communities on all leaves were similar. This result indicates an essential function of taxonomic relatedness in the structure of these communities, i.e., despite the differences observed in the presence and abundance of specific OTUs in earlier samples (by Bray-Curtis), these are phylogenetically related.

The PCoA separating each plant material indicated an apparent clustering based on time (fig S4). No separation based on the treatment (temperature or pH) was observed. It showed that Vibrionales were correlated with the initial samples, and Desulfobacterales and Chromatiales were correlated with samples from the end of the experiment.

The PERMANOVA based on a Bray-Curtis distance matrix indicated that the differences observed over time were significant for all plant species (Table 2). However, only in *L. racemosa*, the temperature had a significant but low effect.

**Table 2:** PERMANOVA test based on Bray-Curtis distance matrix results on the effect of time, temperature and pH on the bacterial community in microcosms of mangrove sediments during leaf litter decomposition

	Pseudo-F	*R*²	P*
Rhizophora mangle			
Time	14.559	0.20345	**0.001**
Temperature	0.674	0.01169	0.855
pH	0.68277	0.01184	0.851
Time: Temperature	0.7969	0.01121	0.647
Time: pH	0.5744	0.00811	0.951
Temperature: pH	0.67255	0.0118	0.828
***Laguncularia racemosa***			
Time	8.6506	0.13591	**0.001**
Temperature	1.8016	0.03172	**0.042**
pH	0.51982	0.00936	0.976
Time: Temperature	2.7612	0.03991	**0.006**
Time: pH	0.5443	0.00869	0.959
Temperature: pH	0.92389	0.01643	0.491
***Avicennia schaueriana***			
Time	18.2720	0.23956	**0.001**
Temperature	1.0450	0.0177	0.372
pH	0.60384	0.0103	0.871
Time: Temperature	1.2835	0.01664	0.183
Time: pH	0.8063	0.01065	0.614
Temperature: pH	0.47727	0.00821	0.986

* - Values in bold indicate the significant differences were found in the tests

## Discussion

In mangrove ecosystems, the organic matter that is not exported through tidal action is deposited on the sediment. Between 35 and 50% of it is soluble and readily available for the microbial community, while the lignocellulosic material is a recalcitrant [3, 29]. The decomposition rate varies due to environmental characteristics and plant composition [29–31]. Thus, the variation observed between plant species can be attributed to the differences in the chemical composition [15, 16].

However, we could only detect a small but not significant effect of pH and temperature on the degradation of this material despite the increase in GHG emissions in the first ten days. Therefore, the rapid loss of weight from the soluble portion of the plant material must be responsible for the GHG emissions, and the lignocellulosic degradation must not be affected by the changes in pH and temperature.

The GHG emission was different between the three sampled species, which indicates that despite a background emission from the sediment, the addition of leaves significantly affected the emission pattern. The differences also agree with previous results that showed that these emissions varied according to the composition of the plant material [32, 33]. In addition, the temperature was one of the parameters that affected GHG production due to a possible increase in the overall metabolic rate of the microorganisms [33], which may lead to positive *feedback*.

The increase in temperature was linked to an increase in the production of CO_2_ likely due to the stimulation of genes involved in decomposition from Proteobacteria, Firmicutes, Actinomycetes, Cyanobacteria and Fungi [34]. In another experiment, they observed an increase in CO_2_ and CH_4_ emissions in flooded soils with a 10 °C increase in the temperature [35]. The link between increasing temperature and GHG emissions is observed due to the differences in temperature between seasons; in summer, the emission is significantly higher in mangroves [9, 36].

The emissions of CH_4_, however, were only higher in *Laguncularia* sp. and *Avicennia* sp. This increase is related to a change in the flow of carbon and electrons, favoring methanogenesis [7, 32, 33, 36]. However, soils and sediments with more alkaline pH usually produce more methane than more acidic ones [33, 36], explaining the contrasts between plants and sampling times.

Despite the differences in CO_2_ and CH_4_ emissions presented above, the production of N_2_O did not differ between samples. The high concentrations of nitrogen in these sediments [4] might explain the contrasting emissions of this gas [7], and the competition with aerobes and sulfate reducers might explain the low emissions observed [37].

The community structure observed here was mainly composed of Proteobacteria, Firmicutes, and Actinobacteria, which is very similar to previous reports [4, 15, 38–42].

The pH is considered a key factor shaping many microbial communities everywhere and has been pinned as central in shaping mangrove microbiomes [39, 42–45]. However, the effects observed in the present study are only minor and focused on an increase in Alpha and Betaproteobacterias in the more acidic treatments, which coincides with the previous assumption that these organisms are indicators of lower pHs and are favored by it [46, 47].

The diversity indexes varied significantly in *Rhizophora* sp. and *Avicennia* sp. with an increase in S_obs_ over time. This increase was proposed to be linked with more recalcitrant compounds such as lignin and cellulose [48]. *Laguncularia* sp. also had a significant increase in diversity with time; however, there was also an effect of temperature. The effect of temperature is similar to previous observations [49] and is related to increased phylogenetic diversity due to a change in the relative abundance of Gammaproteobacteria and Actinobacteria. Despite the known differences in the leaf community composition between plants [18, 50], most of the microorganisms that will colonize after they fall come from the sediment [48].

The process of ecological succession during the decay of these leaves follows a clear pattern. Although there was a clear separation between plants and sampling time, there was no separation between different pH and temperatures. However, there was an evident effect on OTUs from Chromatiales, Clostridiales, and mainly Desulfobacterales during decomposition. Desulfobacterales are sulphate reducers commonly observed in mangroves microbiomes [4, 6, 38, 40, 51].

The changes observed in our experiment indicate that even with climate change, the critical factor for colonisation, degradation and GHG emissions is the plant species. Probably due to the chemical composition of the material. The changes in pH and temperature did not significantly influence the microbial community structure and degradation rate. However, temperature changes have increased the emission of CO_2_ and CH_4_ in the first ten days of decomposition. Altogether, our data show that even though the microbial community structure in these decaying leaves was not affected, its activity was, and this alteration was likely related to the decomposition of soluble non-recalcitrant compounds. This highlights the complexity of the responses of microorganisms to the slight changes predicted to occur and that they can have significant global impacts if it happens equally throughout the mangroves.

In conclusion, our study shows role of environmental parameters in shaping greenhouse gas (GHG) emissions during the decomposition of organic matter in mangrove ecosystems. While we observed that pH and temperature changes had minor effects on the microbial community structure and overall degradation rates, they did influence the activity of these communities, particularly in the early stages of decomposition. The rapid decomposition of soluble plant materials was a significant driver of initial CO_2_ and CH_4_ emissions, which varied among different plant species. Furthermore, although temperature increases correlated with higher CO_2_ emissions, reflecting a rise in microbial metabolic rates, the overall impact of pH and temperature on microbial community structure was limited. Our findings highlight the nuanced and complex responses of microbial communities to environmental changes, suggesting that even slight alterations in climate parameters can significantly impact GHG emissions in mangrove ecosystems which could lead to greater warming and acidification. These insights are crucial for understanding the broader implications of climate change on these coastal systems and of the predicted climate scenarios for the GHG emissions from mangrove forests.

## Electronic supplementary material

Below is the link to the electronic supplementary material.

Supplementary Material 1

## References

[1] Siikamäki J, Sanchirico JN, Jardine SL (2012) Global economic potential for reducing carbon dioxide emissions from mangrove loss. Proc Natl Acad Sci USA 109(36):14369–14374. 10.1073/pnas.1200519109 22847435 10.1073/pnas.1200519109 PMC3437861

[2] Alongi DM (2014) Carbon Cycling and Storage in Mangrove forests. Annual Rev Mar Sci 6(1):195–219. 10.1146/annurev-marine-010213-135020 10.1146/annurev-marine-010213-135020 24405426

[3] Kristensen E, Bouillon S, Dittmar T, Marchand C (2008) Organic carbon dynamics in mangrove ecosystems: a review. Aquat Bot 89(2):201–219. 10.1016/j.aquabot.2007.12.005

[4] Andreote F, DJ J, ACF DC, DM D (2012) The Microbiome of Brazilian Mangrove sediments as revealed by Metagenomics. PLoS ONE 7(6):e38600. 10.1371/journal.pone.0038600 22737213 10.1371/journal.pone.0038600 PMC3380894

[5] Allen D, Dalal RC, Rennenberg H, Schmidt S (2011) Seasonal variation in nitrous oxide and methane emissions from subtropical estuary and coastal mangrove sediments, Australia. Plant Biol (Stuttgart Germany) 13(1):126–133. 10.1111/j.1438-8677.2010.00331.x 10.1111/j.1438-8677.2010.00331.x 21143733

[6] Taketani RG, Yoshiura CA, Dias ACF, Andreote FD, Tsai SM (2010) Diversity and identification of methanogenic archaea and sulphate-reducing bacteria in sediments from a pristine tropical mangrove. Antonie Van Leeuwenhoek 97(4):401–411. 10.1007/s10482-010-9422-8 20195901 10.1007/s10482-010-9422-8

[7] Konnerup D, Betancourt-Portela JM, Villamil C, Parra JP (2014) Nitrous oxide and methane emissions from the restored mangrove ecosystem of the Ciénaga Grande De Santa Marta, Colombia. Estuar Coast Shelf Sci 140:43–51. 10.1016/j.ecss.2014.01.006

[8] Purvaja R, Ramesh R, Frenzel P (2004) Plant-mediated methane emission from an Indian mangrove: PLANT-MEDIATED METHANE EMISSION FROM AN INDIAN MANGROVE. Glob Change Biol 10(11):1825–1834. 10.1111/j.1365-2486.2004.00834.x

[9] Chen GC, Tam NFY, Ye Y (2012) Spatial and seasonal variations of atmospheric N2O and CO2 fluxes from a subtropical mangrove swamp and their relationships with soil characteristics. Soil Biol Biochem 48:175–181. 10.1016/j.soilbio.2012.01.029

[10] Liu J, Zhou Y, Valach A et al (2020) Methane emissions reduce the radiative cooling effect of a subtropical estuarine mangrove wetland by half. Glob Change Biol 26(9):4998–5016. 10.1111/gcb.15247 10.1111/gcb.15247 32574398

[11] Parry ML (2007) Climate Change 2007-Impacts, adaptation and vulnerability: Working Group II Contribution to the Fourth Assessment Report of the IPCC, vol 4. Cambridge University Press

[12] Webber M, Calumpong H, Ferreira B et al Mangroves. Chapter 48. The First Global Integrated Marine Assessment World Ocean Assessment I by the Group of Experts of the Regular Process. *Lorna Inniss and Alan Simcock (Joint Coordinators) United Nations General Assembly and its Regular Process for Global Reporting and Assessment of the State of the Marine Environment, including Socioeconomic Aspects*. Published online 2016

[13] Alongi DM (2015) The impact of Climate Change on Mangrove forests. Curr Clim Change Rep 1(1):30–39. 10.1007/s40641-015-0002-x

[14] Moitinho MA, Chiaramonte JB, Bononi L, Gumiere T, Melo IS, Taketani RG (2022) Fungal succession on the decomposition of three plant species from a Brazilian mangrove. Sci Rep 12(1):14547. 10.1038/s41598-022-18667-x 36008524 10.1038/s41598-022-18667-x PMC9411622

[15] Moitinho MA, Bononi L, Souza DT, Melo IS, Taketani RG Bacterial Succession Decreases Network Complexity During Plant Material Decomposition in Mangroves. *Microbial Ecology*. Published online 2018. 10.1007/s00248-018-1190-4 10.1007/s00248-018-1190-4 29687224

[16] Taketani RG, Moitinho MA, Mauchline TH, Melo IS (2018) Co-occurrence patterns of litter decomposing communities in mangroves indicate a robust community resistant to disturbances. PeerJ 6:e5710. 10.7717/peerj.5710 30310750 10.7717/peerj.5710 PMC6174875

[17] Benton TG, Solan M, Travis JMJ, Sait SM (2007) Microcosm experiments can inform global ecological problems. Trends Ecol Evol 22(10):516–521. 10.1016/j.tree.2007.08.003 17822805 10.1016/j.tree.2007.08.003

[18] Moitinho MA, Chiaramonte JB, Souza DT et al (2019) Intraspecific variation on epiphytic bacterial community from Laguncularia racemosa phylloplane. Brazilian J Microbiol 50(4). 10.1007/s42770-019-00138-7 10.1007/s42770-019-00138-7 PMC6863213 31473927

[19] Stocker TF, Qin D, Plattner GK et al Climate Change 2013: The physical science basis. contribution of working group I to the fifth assessment report of IPCC the intergovernmental panel on climate change. Published online 2014

[20] Dickson AG, Sabine CL, Christian JR (2007) Guide to Best practices for Ocean CO2 measurements. North Pacific Marine Science Organization

[21] European Commission. Directorate-General for Research. Guide to Best Practices for Ocean Acidification Research and Data Reporting. Publications Office (2010) Accessed June 9, 2021. https://data.europa.eu/doi/10.2777/58454

[22] Olson JS (1963) Energy Storage and the balance of producers and decomposers in Ecological systems. Ecology 44(2):322–331. 10.2307/1932179

[23] Jantalia CP, dos Santos HP, Urquiaga S, Boddey RM, Alves BJ (2008) Fluxes of nitrous oxide from soil under different crop rotations and tillage systems in the South of Brazil. Nutr Cycl Agrosyst 82(2):161–173

[24] Sogin ML, Morrison HG, Huber JA et al (2006) Microbial diversity in the deep sea and the underexplored rare biosphere. Proc Natl Acad Sci USA 103(32):12115–12120 16880384 10.1073/pnas.0605127103 PMC1524930

[25] Wang Y, Qian PY (2009) Conservative fragments in bacterial 16S rRNA genes and primer design for 16S ribosomal DNA amplicons in metagenomic studies. PLoS ONE 4(10):e7401. 10.1371/journal.pone.0007401 19816594 10.1371/journal.pone.0007401 PMC2754607

[26] Caporaso JG, Kuczynski J, Stombaugh J et al (2010) QIIME allows analysis of high- throughput community sequencing data. Nat Methods 7(5):335–336. 10.1038/nmeth0510-335 20383131 10.1038/nmeth.f.303 PMC3156573

[27] Mcmurdie PJ, Holmes S (2013) Phyloseq: an R Package for Reproducible Interactive Analysis and Graphics of Microbiome Census Data. 8(4). 10.1371/journal.pone.0061217 10.1371/journal.pone.0061217 PMC3632530 23630581

[28] Oksanen J, Blanchet FG, Friendly M et al vegan: Community Ecology Package. Published online 2018. https://cran.r-project.org/package=vegan

[29] Benner R, Hodson R (1985) Microbial degradation of the leachable and lignocellulosic components of leaves and wood from Rhizophora mangle in a tropical mangrove swamp. Mar Ecol Prog Ser 23:221–230. 10.3354/meps023221

[30] Gessner MO, Chauvet E, Dobson M (1999) A perspective on Leaf Litter Breakdown in streams. Oikos 85(2):377. 10.2307/3546505

[31] Xing Y, Qiu J, Chen J et al (2024) Unveiling hidden interactions: microorganisms, enzymes, and mangroves at different stages of succession in the Shankou Mangrove Nature Reserve, China. Sci Total Environ 923:171340. 10.1016/j.scitotenv.2024.171340 38438047 10.1016/j.scitotenv.2024.171340

[32] Nóbrega GN, Ferreira TO, Siqueira Neto M et al (2016) Edaphic factors controlling summer (rainy season) greenhouse gas emissions (CO2 and CH4) from semiarid mangrove soils (NE-Brazil). Sci Total Environ 542:685–693. 10.1016/j.scitotenv.2015.10.108 26546764 10.1016/j.scitotenv.2015.10.108

[33] Oertel C, Matschullat J, Zurba K, Zimmermann F, Erasmi S (2016) Greenhouse gas emissions from soils—A review. Geochemistry 76(3):327–352. 10.1016/j.chemer.2016.04.002

[34] Wang H, He Z, Lu Z et al (2012) Genetic linkage of Soil Carbon pools and microbial functions in Subtropical Freshwater wetlands in response to experimental warming. Appl Environ Microbiol 78(21):7652–7661. 10.1128/AEM.01602-12 22923398 10.1128/AEM.01602-12 PMC3485724

[35] Brooker MR, Bohrer G, Mouser PJ (2014) Variations in potential CH4 flux and CO2 respiration from freshwater wetland sediments that differ by microsite location, depth and temperature. Ecol Eng 72:84–94. 10.1016/j.ecoleng.2014.05.028

[36] Hu M, Ren H, Ren P, Li J, Wilson BJ, Tong C (2017) Response of gaseous carbon emissions to low-level salinity increase in tidal marsh ecosystem of the Min River estuary, southeastern China. J Environ Sci 52:210–222. 10.1016/j.jes.2016.05.009 10.1016/j.jes.2016.05.009 28254041

[37] Alongi DM, Wattayakorn G, Pfitzner J et al (2001) Organic carbon accumulation and metabolic pathways in sediments of mangrove forests in southern Thailand. Mar Geol 179(1):85–103. 10.1016/S0025-3227(01)00195-5

[38] Dias ACF, Andreote FD, Rigonato J, Fiore MF, Melo IS, Araújo WL (2010) The bacterial diversity in a Brazilian non-disturbed mangrove sediment. Antonie Van Leeuwenhoek 98(4):541–551. 10.1007/s10482-010-9471-z 20563848 10.1007/s10482-010-9471-z

[39] Mendes LW, Tsai SM Variations of Bacterial Community structure and composition in Mangrove sediment at different depths in Southeastern Brazil. Published Online 2014:827–843. 10.3390/d6040827

[40] Varon-Lopez M, Dias ACF, Fasanella CC et al (2014) Sulphur-oxidizing and sulphate-reducing communities in Brazilian mangrove sediments. Environ Microbiol 16(3):845–855. 10.1111/1462-2920.12237 24033859 10.1111/1462-2920.12237

[41] Basak P, Pramanik A, Roy R, Chattopadhyay D, Bhattacharyya M (2015) Cataloguing the bacterial diversity of the Sundarbans mangrove, India in the light of metagenomics. Genomics Data 4:90–92. 10.1016/j.gdata.2015.03.014 26484187 10.1016/j.gdata.2015.03.014 PMC4535861

[42] Wu P, Xiong X, Xu Z et al (2016) Bacterial communities in the rhizospheres of three Mangrove Tree species from Beilun Estuary, China. Yan Z Guang. ed PLoS ONE 11(10):e0164082. 10.1371/journal.pone.0164082 10.1371/journal.pone.0164082 PMC5047532 27695084

[43] Liu S, Ren H, Shen L et al (2015) pH levels drive bacterial community structure in sediments of the Qiantang River as determined by 454 pyrosequencing. Front Microbiol 6. 10.3389/fmicb.2015.00285 10.3389/fmicb.2015.00285 PMC4403504 25941515

[44] Chen Q, Zhao Q, Li J, Jian S, Ren H (2016) Mangrove succession enriches the sediment microbial community in South China. Sci Rep 6(1):27468. 10.1038/srep27468 27265262 10.1038/srep27468 PMC4893734

[45] Bai R, Wang JT, Deng Y, He JZ, Feng K, Zhang LM (2017) Microbial Community and functional structure significantly varied among distinct types of Paddy Soils but responded differently along gradients of soil depth layers. Front Microbiol 8:945. 10.3389/fmicb.2017.00945 28611747 10.3389/fmicb.2017.00945 PMC5447084

[46] Krause E, Wichels A, Giménez L, Lunau M, Schilhabel MB, Gerdts G (2012) Small Changes in pH Have Direct Effects on Marine Bacterial Community Composition: A Microcosm Approach. Kirchman DL, ed. *PLoS ONE*.;7(10):e47035. 10.1371/journal.pone.0047035 10.1371/journal.pone.0047035 PMC3469576 23071704

[47] Lv X, Ma B, Yu J et al (2016) Bacterial community structure and function shift along a successional series of tidal flats in the Yellow River Delta. Sci Rep 6(1):36550. 10.1038/srep36550 27824160 10.1038/srep36550 PMC5099912

[48] Xu W, Shi L, Chan O, Li J, Casper P, Zou X (2013) B Stevenson ed. Assessing the Effect of Litter Species on the Dynamic of Bacterial and Fungal communities during Leaf Decomposition in Microcosm by Molecular techniques. PLoS ONE 8 12 e84613 10.1371/journal.pone.0084613 24367682 10.1371/journal.pone.0084613 PMC3868619

[49] Cadotte MW, Jonathan Davies T, Regetz J, Kembel SW, Cleland E, Oakley TH (2010) Phylogenetic diversity metrics for ecological communities: integrating species richness, abundance and evolutionary history. Ecol Lett 13(1):96–105. 10.1111/j.1461-0248.2009.01405.x 19903196 10.1111/j.1461-0248.2009.01405.x

[50] Dias ACF, Taketani RG, Andreote FD et al Interespecific variation of the bacterial community structure in the phyllosphere of mangrove forests. Brazilian J Microbiol Published Online 2012:653–660 10.1590/S1517-83822012000200030 PMC3768840 24031877

[51] Ranjan Mishra R, Ranjan Swain M, Kanti Danga T, Thatoi H (2012) Diversity and seasonal fluctuation of predominant microbial communities in Bhitarkanika, a tropical mangrove ecosystem in India. Revista De biología Trop 60(2):909–924 10.15517/rbt.v60i2.4026 23894955

