# Supplementary material for: Organic matter decay and bacterial community succession in mangroves under simulated climate change scenarios
Source: Braz J Microbiol. 2024 Jul 19;55(4):3353–62. doi: 10.1007/s42770-024-01455-2 (PMC11711568; doi:10.1007/s42770-024-01455-2)
Supplement: Supplementary file 1 — Supplementary Material 1 [file 42770_2024_1455_MOESM1_ESM.docx]

**Table S1. Comparison of the flux of emission of CO_2_ and CH_4_** in microcosms of mangrove sediments during leaf litter during 45 days of degradation. Incubated under different pHs and temperature.

| **Incubation condition** | **Average emission flow of CO_2_ (μg C cm^-2^ day^-1^)** | | | | |
| --- | --- | --- | --- | --- | --- |
|  | *R. mangle* | *L. racemosa* | *A. schaueriana* | | Control |
| **29.5ºC** | 580.46^a^ | 489.92*^a^* | 551.37**^A^** | -177.10^a’^ | |
| **27.5ºC** | 823.18^a^ | 275.37*^a^* | 248.09**^B^** | 156.16^b’^ | |
| **pH 7.05** | 582.29**^a^** | 380.75^A^ | 381.16*^A^* | -2.57***^a’^*** | |
| **pH 6.74** | 821.35**^a^** | 384.53^A^ | 418.3*^A^* | -18.56***^a’^*** | |
|  | **Average emission flow of CH_4_ (μg CO_2eq._cm^-2^ day^-1^)** | | | | |
|  | *R. mangle* | *L. racemosa* | *A. schaueriana* | Control | |
| **29.5ºC** | 183.86*^a^* | 59.13***^A^*** | 345.16^a^ | -46.75^a’^ | |
| **27.5ºC** | 203.89*^a^* | 9.94***^B^*** | 126.33^b^ | -13.94^b’^ | |
| **pH 6.74** | 195.81^A^ | 38.40*^A^* | 282.75**^a^** | -26.52***^a’^*** | |
| **pH 7.05** | 191.81^A^ | 30.67*^A^* | 188.74**^a^** | -34.17***^a’^*** | |

*different letters over each value indicate significant differences in Tukey’s HSD test with α 0.05. capital letters indicate differences between different pH treatment and regular letters indicate differences between temperature treatments.


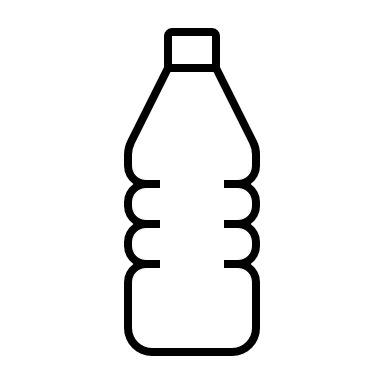


**Figure S1: Microcosm bottle diagram.**

**Figure S2. Comparison of the results of diversity indexes obtained** from the bacterial community 16S rRNA gene sequencing obtained from in microcosms of mangrove sediments during leaf litter decomposition. Samples are color coded according to the legend (**a.) Observed** OTUs; (**b.**) Richness estimator Chao1; (**c.**) Shannon’s H’ diversity index e (**d.**) Faith’s phylogenetic diversity (PD). Letters on top of samples indicate a significant difference in Tukey’s HSD with an α of 0.05

**Figure S3.** Composition of the bacterial community 16S rRNA gene sequencing obtained from in microcosms of mangrove sediments during leaf litter decomposition. The Y-axis represents the number of reads after rarefication to 40.000 reads per sample.

**Figure S4.** Principal coordinate analysis (PCoA) based on a distance matrix of Bray-Curtis of the bacterial community of decaying leaves of *Rhizophora mangle*. Samples are coded according to (**a.**) Time and order (OTU plot); (**b.**) temperature e (**c.**) pH.
